# A novel approach for enhancing the color and antimicrobial properties of pine and beech wood using Se-NPs

**DOI:** 10.1038/s41598-023-39748-5

**Published:** 2023-08-10

**Authors:** Tarek Abou Elmaaty, Abeer Swidan, Khaled Sayed-Ahmed, Nancy Zaghloul

**Affiliations:** 1 Department of Textile Printing, Dyeing and Finishing, Faculty of Applied Arts, Damietta University, Damietta, 34512 Egypt; 2 Department of Interior Design and Furniture, Faculty of Applied Arts, Damietta University, Damietta, 34512 Egypt; 3 Department of Agricultural Biotechnology, Faculty of Agriculture, Damietta University, Damietta, 34512 Egypt

**Keywords:** Chemical biology, Microbiology, Environmental sciences, Chemistry, Materials science, Nanoscience and technology

## Abstract

Pine wood (PW) and beech wood (BW) are the most used wood in furniture and other applications owing to their unique characteristics and low machining cost. However, their biodegradability and varied moisture content limit their wider use and durability. Therefore, in this study, nanotechnology was used as a novel eco-friendly approach to enhance the durability, antimicrobial properties, and color of wood. Selenium nanoparticles (Se-NPs) were prepared in spherical shape at varied concentrations (25 and 50 mM) using an eco-friendly method in the range of 35–80 and 40–155 nm, respectively. Se-NPs formation at the nanoscale was confirmed using UV/Vis analysis, transmission electron microscopy (TEM), and X-ray diffraction (XRD). The prepared Se-NPs were then impregnated into PW and BW for different periods ranging from 2 h to 1 week. The treated wood were then leached in distilled water for 14 days to eliminate excess Se-NPs from the wood surface. The treated wood surfaces were examined using energy-dispersive X-ray spectroscopy (EDX) and scanning electron microscopy (SEM). In addition, the depth of Se-NPs penetration into the treated wood at both tangential and radial sides was determined. Se-NPs impacts on the color properties, density, moisture content and antimicrobial activities of the treated wood were evaluated. PW treated with Se-NPs showed better antimicrobial and color characteristics than treated BW. PW samples immersed in 50 mM Se-NPs for 2 h showed the highest K/S values, whereas the highest antimicrobial values were obtained for those immersed at the same concentration for 2 days, and 1 week.

## Introduction

Wood has been used for centuries for many reasons owing to its outstanding qualities. It represents a primary raw material because of its high strength, low weight, and relative durability. Therefore, it can be used in numerous applications, such as indoor and outdoor applications, if treated with efficient materials^1,2^. All wood are derived from trees that are either softwood or hardwood, according to botanical classification, such as pine (*Pinus sylvestris*) and beech (*Fagus sylvatica*)^3,4^. PW is used in furniture owing to its good strength-to-weight ratio; and therefore, it is typically regarded as appealing wood^5^. BW is a sturdy wood that machines well and is ideal for steam bending^6^. In addition, it is a reasonably priced material with a low machining cost^7^. However, there are two disadvantages that minimize mainly its wider use and durability, including biodegradability and dimensional instability as a result of change in its moisture content^8–10^. Additionally, the traditional wood treatments, including paints, stains, varnishes, polishes, and adhesives, if not handled properly, can harm the environment and humans^11^.

In this respect, the use of nanotechnology can enhance the durability of wood, thereby increasing the service lifetime of the wood products such as furniture due to the unique properties of NPs in the range of 100 nm or less^12^. The concepts of biological, physical, material, and chemical sciences are merging in nanotechnology for the development of various technologies^13^. When using different NPs for wood protection, it is possible to reduce moisture uptake and improve ultraviolet protection, mechanical properties, and fire resistance^14–17^. NPs provide a wide variety of antimicrobial classes, and offer persistent antibacterial action with little toxicity^18^. In addition, they have the ability to impart multifunctional properties and coloration to materials without compromising the inherent characteristics of the substrate^19,20^. A wide range of color tunability is possible owing to the optical properties of NPs such as surface plasmon resonance, quantum confinement effects, and NPs-structured colors. By changing size, shape, composition, and surface function, NPs could have different colors^21,22^.

Novel wood composites with improved characteristics have been developed with the help of nanotechnology^23^. Wood plastic composites with improved physical, mechanical, and thermal properties can be created by adding nanofillers such as ZnO-NPs, TiO_2_-NPs, nanoclays, and SiO_2_-NPs^24–26^. It is possible to produce plywood composites with improved flexural strength, dimensional stability, bonding strength, flexural modulus, and screw withdrawal resistance properties using SiO_2_-NPs, Al_2_O_3_-NPs, and ZnO-NPs^27^. In addition, the modulus of rupture, modulus of elasticity, bonding strength, and screw withdrawal resistance of the particleboard composites were enhanced by reinforcement with SiO_2_-NPs and Al_2_O_3_-NPs^28^. Because of their carbon-neutral structure, low toxicity, biodegradability, wide range availability, and superior properties, lignocellulosic green nanomaterials have great potential for fabricating wood plastic composites with improved characteristics^24^. Eco-friendly wood-based composite panels can be fabricated using low-formaldehyde-emitting adhesives enhanced by the addition of nanocellulose at proper loading levels^23,29^ and using SiO_2_-NPs, Al_2_O_3_-NPs, and ZnO-NPs^30^.

Green nanotechnology refers to the synthesis of NPs without hazard chemicals to limit the cytotoxicity levels of the prepared NPs^31–33^. Biomolecules and eco-friendly substances have been discovered to have an obvious role in the production of NPs of all shapes and sizes, paving the way for the development of greener, and safer NPs synthesis techniques^34–36^. Selenium is a non-metallic element, it is a trace micronutrient element that is extremely important in the ecosystem^37,38^. Se-NPs with a red hue represent a novel study target because of their unique properties, low toxicity relative to selenium molecules, and outstanding bioactivity^39,40^. Due to their antimicrobial properties, Se-NPs are becoming increasingly important in the food and medical industries^41^. However, its practical features as a wood treatment to improve the physicochemical characteristics of wood and give it an aesthetic color have yet to be described.

Thus, the objective of this study was to produce sustainable materials for furniture production using nanotechnology. In this respect, green synthesized Se-NPs were impregnated into PW and BW to enhance their durability, antimicrobial properties and color characteristics. Se-NPs were used at different concentrations and impregnation periods to evaluate the effects of concentration and impregnation time on the antimicrobial and color properties of the treated wood compared to those of the control.

## Experimental sections

### Materials

Two different types of wood, non-defective sapwood of pine (*Pinus sylvestris L.*) and beech (*Fagus sylvatica L.*), were purchased from Moelven (Hedmark, Norway) and CEDAR d.o.o. (Rijeka, Croatia), respectively. The collection of the studied wood complied with the relevant institutional, national, and international guidelines and legislation. Wood specimens were then used with dimensions of 25 × 25 × 15 mm^3^ and were sorted into eight groups of PW and BW for different impregnation periods of 2 hrs, 1 day, 2 days, and 1 week (each group consisted of 10 specimens). Sodium hydrogen selenite, polyvinylpyrrolidone (PVP), and ascorbic acid were obtained from Sigma-Aldrich and were used without further purification.

### Procedures

#### Green synthesis of Se-NPs

Se-NPs were synthesized via redox reaction based on the procedure described by Abou Elmaaty *et al*.^42^. Sodium hydrogen selenite was utilized, as a precursor for Se-NPs at different concentrations of 50 and 100 mM. Polyvinylpyrrolidone (PVP) was then added to this solution at a concentration of 12 g/100 ml to maintain the stability of the prepared Se-NPs. In addition, ascorbic acid at various concentrations (100 mM and 200 mM) was added to the previous mixture at a molar ratio of 2:1 and a volume ratio of 1:1 (vitamin C: sodium hydrogen selenite). The formation of Se-NPs was confirmed after the change in solution color from colorless to dark orange^43^. For XRD analysis, the Se-NPs colloidal solution was completely dried at 130 °C and then stored at 4 °C for further use.

#### Impregnation of Se-NPs into wood and leaching.

**Figure 1:**
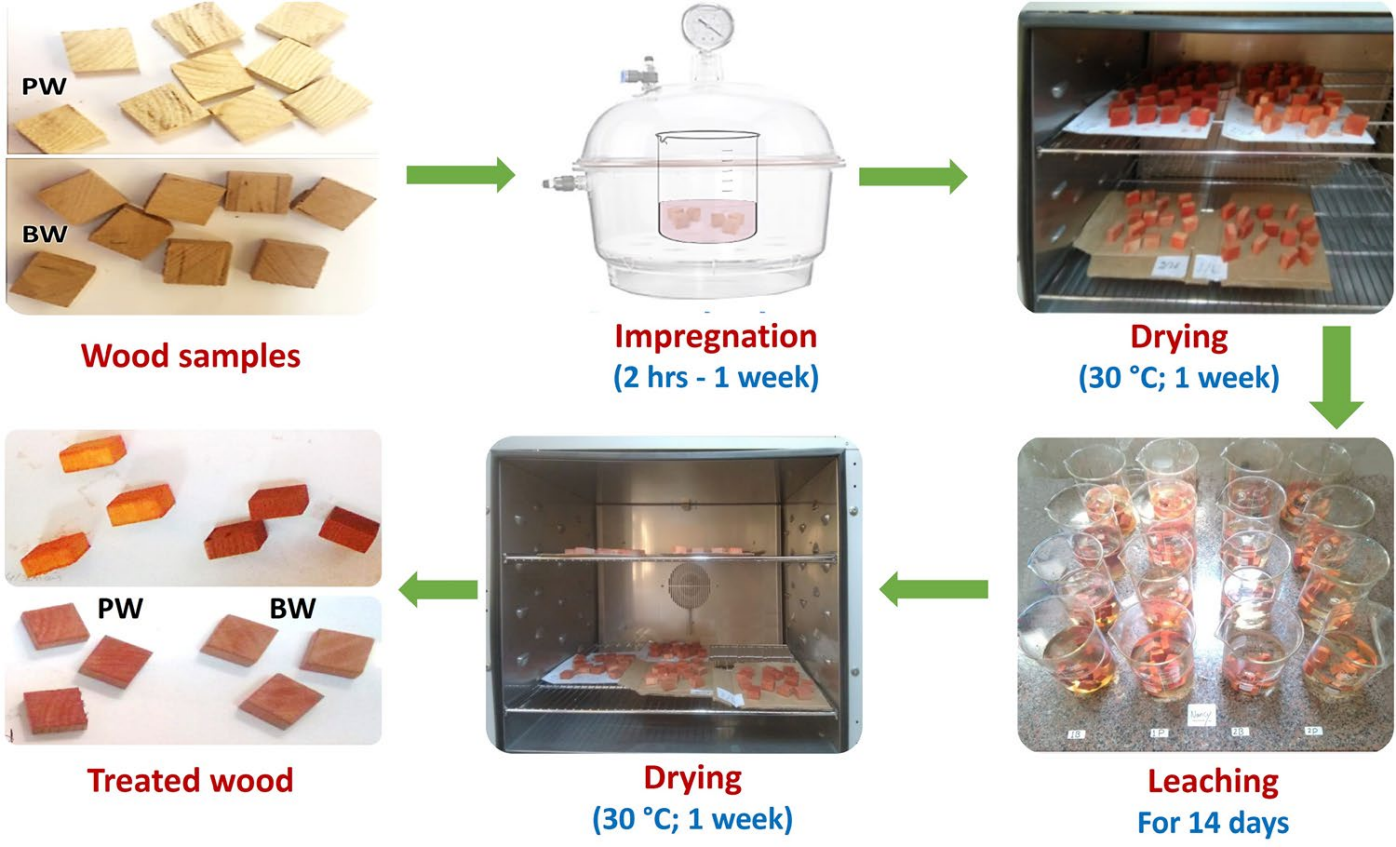
Schematic drawing of the impregnation process of PW and BW with Se-NPs.

PW and BW specimens were impregnated with Se-NPs colloidal solutions at concentrations of 25mM and 50mM inside a vacuum desiccator at a vacuum of 80 kPa. The impregnation process was carried out for various periods of 2 hrs, 1 day, 2 days, and 1 week to study the effect of impregnation time on the color and antimicrobial properties of the tested wood samples. Wood samples treated with Se-NPs were then dried at 30 °C in an oven for one week. Then, the treated samples were leached. In this respect, the equilibrium moisture content was attained by conditioning the treated specimens at 20 °C and 65% RH. The specimens were then immersed in distilled water (20 °C, pH 5.5) for 14 days. The aim of the leaching process was to remove excess Se-NPs that were not well deposited onto the wood surfaces. The water was changed four times during the leaching process: after 2 hrs, 2 days, 4 days, and 8 days of leaching^44^. The leached wood specimens were then dried in an oven at 30 °C for 1 week, as shown in Figure 1.

### Characterization of Se-NPs and wood surface

#### TEM analysis

Se-NPs were examined using a transmission electron microscope (JEOL, JEM 2100F, Tokyo, Japan) at 200 kV to characterize their morphology and size. A drop of Se-NPs solution was added to a copper grid (400-mesh) coated with carbon, and the solvent was then let in air at room temperature until its evaporation.

#### UV–Vis spectroscopy

Se-NPs formation was also qualitatively confirmed via UV/Vis spectra of Se-NPs colloidal solutions due to their surface plasmon resonance (SPR) using UV-visible spectrophotometer (Shimadzu Co., Kyoto, Japan).

#### X-ray diffraction (XRD)

Xray diffractometer (Bruker D8 ADVANCE, Karlsruhe, Germany) was used to determine the crystalline nature of the synthesized Se-NPs and those absorbed on the treated wood surfaces.

### Testing of the treated specimens

#### SEM and EDX analyses

Wood samples were examined using a scanning electron microscope (JEOL JSM-6510LB, Tokyo, Japan) to study the effect of impregnation with Se-NPs on the morphology of their surfaces, compared to blank samples. In addition, chemical analysis of the elements found on the wood surface was conducted using an energy dispersive spectroscopy (EDX) unit attached to a scanning electron microscope.

#### Colorimetric analysis

K/S values and color characteristics of the treated PW and BW specimens were measured using spectrophotometer (Minolta CM-3600 d, Tokyo, Japan), and then compared with the untreated samples as a control.

#### Determination of moisture content, density, and penetration depth

The moisture content and density of the tested wood samples were determined according to ISO 13061-1:2014^45^ and ISO 13061-2:2014^46^, respectively. The depth of Se-NPs penetration into the treated wood at both tangential and radial sides was measured using a digital vernier caliper (silverline, UK).

#### Evaluation of antimicrobial activities of wood specimens

The antimicrobial activities of the blank wood specimens or those treated with Se-NPs against G-ve bacteria (*Escherichia coli*), G+ve bacteria (*Bacillus cereus* and *Staphylococcus aureus*), and yeast (*Candida albicans*) were tested based on the AATCC Test Method (147-1988) and the zone of growth inhibition (mm) was used as an expression for the antimicrobial activity^47^.

#### Statistical analysis

The data obtained from this study were statistically analyzed using the Costat program version 6.311 (CoHort software, Monterey, USA). Wood samples were compared using statistical analysis of variance (ANOVA). In addition, the standard deviation (SD) of the obtained data was calculated. Duncan’s new range test at P = 0.05 was used to determine the significant variations among all means. Each sample in this study was analyzed three times^48–50^.

## Results and discussion

### Characterization of synthesized Se-NPs

#### TEM analysis

**Figure 2:**
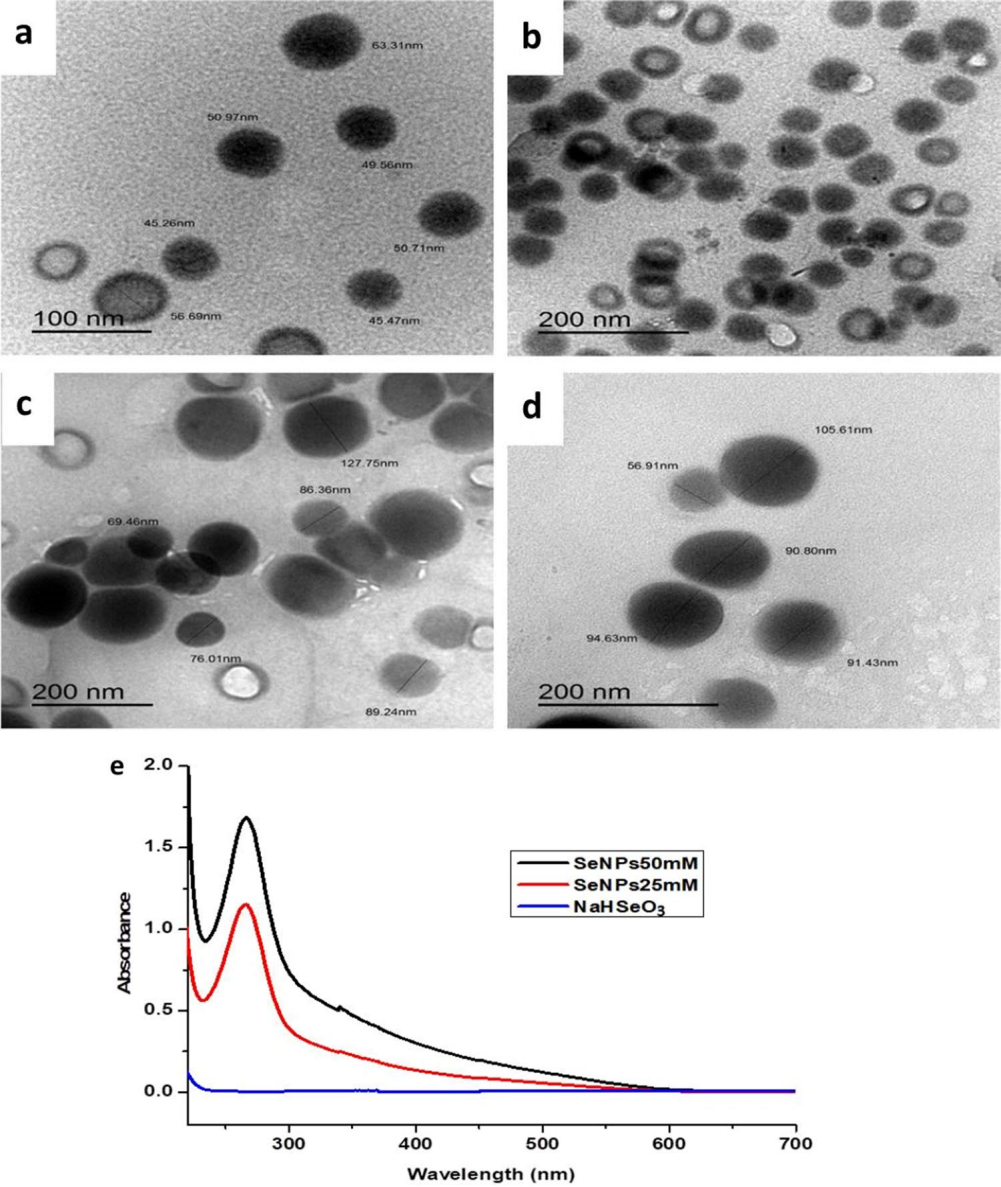
TEM images of Se-NPs at different concentrations of (**a**,**b**) 25 mM and (**c**,** d**) 50 mM, and UV/Vis spectra of Se-NPs synthesized at various concentrations and sodium hydrogen selenite.

The prepared Se-NPs were characterized using transmission electron microscopy (TEM) to study the effect of the Se-NPs concentration on their morphology and size. TEM micrographs confirmed the formation of well-dispersed spherical Se-NPs, as shown in Figure 2. Moreover, these micrographs showed no deformation or aggregation in the colloidal solution of the synthesized Se-NPs. Most of the NPs prepared at a concentration of 25 mM were in the range of 35-80 nm. On the other hand, the majority of Se-NPs at a concentration of 50 mM showed larger diameters ranged from 40 to 155 nm, illustrating that Se-NPs size increased as the increase in NPs concentration.

Furthermore, the Se-NPs prepared at a concentration of 25 mM were spherical and ring-shaped. Whereas, ordinary solid spherical particles were observed for Se-NPs synthesized at a concentration of 50 mM. The micrographs obtained from TEM analysis revealed that the specific surface area of Se-NPs increased with the reduction in NPs concentration owing to the decrease in the Se-NPs diameters and their hollow shape, as displayed in Figure 2.

#### UV–Vis spectroscopy

Se-NPs formation was confirmed by the change in the color of Se-NPs colloidal solution from colorless to dark orange due to the surface plasmon resonance phenomenon (SPR) as a result of the electrons combined vibrations of the obtained Se-NPs^51^. UV-Vis spectra ranging from 200 to 700 nm were utilized for Se-NPs characterization at varied concentrations (25 mM and 50 mM). The Se-NPs exhibited a maximum peak at approximately 266 nm, confirming the formation of Se-NPs in spherical shape^52^, as displayed in Figure 2 (e).

### Testing of the wood treated with Se-NPs

#### SEM and EDX analysis

**Figure 3:**
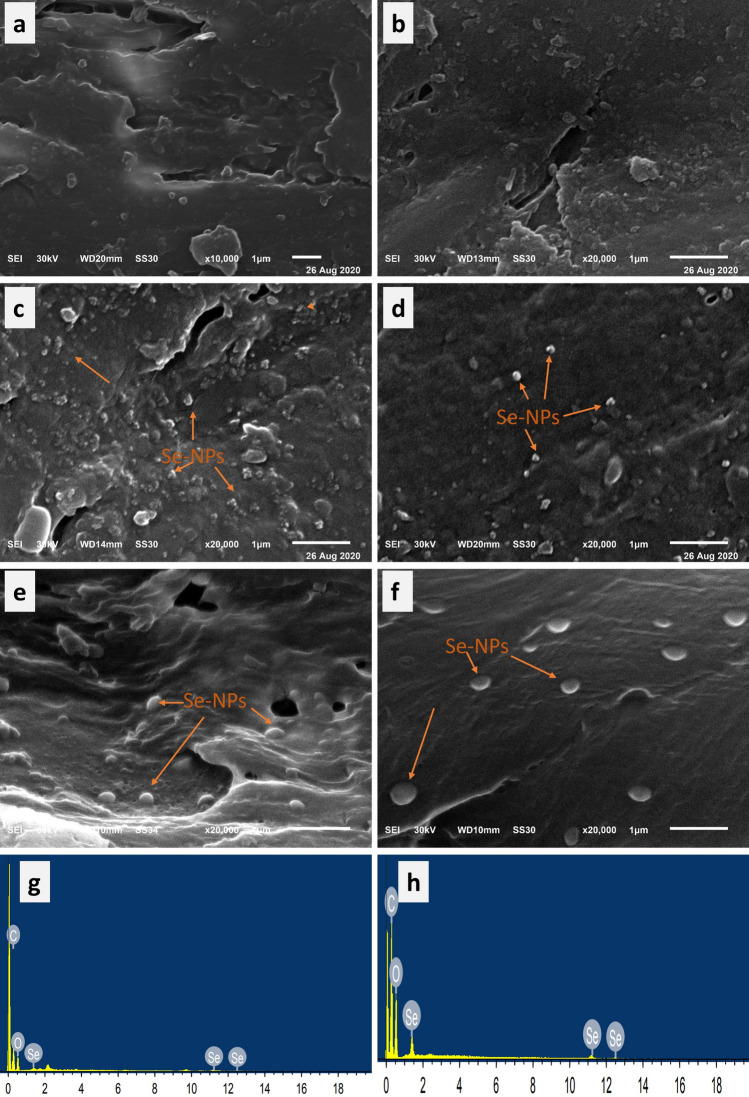
SEM images of untreated (**a**) PW, (**b**) BW, the treated (**c**) PW, (**d**) BW with 50 mM Se-NPs at transverse section, the treated (**e**) PW, (**f**) BW with 50 mM Se-NPs at tangential side, and (**g**,** h**) EDX spectra of treated PW and BW.

SEM analysis was carried out to confirm the deposition of the synthesized Se-NPs on the treated wood surfaces at both transverse and tangential sides, comparing to the reference samples of untreated PW and BW. SEM micrographs of the blank wood specimens showed that their surfaces were typically clear with scales free from Se-NPs. An obvious change in the morphology of wood specimens surfaces after the deposition of Se-NPs on them. The surfaces of PW and BW treated with Se-NPs exhibited shiny and spherical particles at the nanoscale, indicating the presence of Se-NPs on the surfaces of the treated wood, as displayed in Figure 3. SEM micrographs illustrated that Se-NPs were well distributed on the treated wood surfaces impregnated with Se-NPs. In addition, more Se-NPs were deposited on the treated PW surface than on the BW surface. PW exhibits broader pores and higher permeability than BW that enables it to absorb larger quantities of Se-NPs than BW^15^. The EDX spectra of PW and BW treated with Se-NPs were obtained to analyze the chemical elements found on their surfaces. The EDX spectra of PW and BW impregnated with Se-NPs showed peaks corresponding to Se-NPs at approximately 1.3, 11.2 and 12.5 Kev, confirming the successful deposition of Se-NPs on their surfaces^53^, as displayed in Figure 3 (e,f).

#### XRD analysis

**Figure 4:**
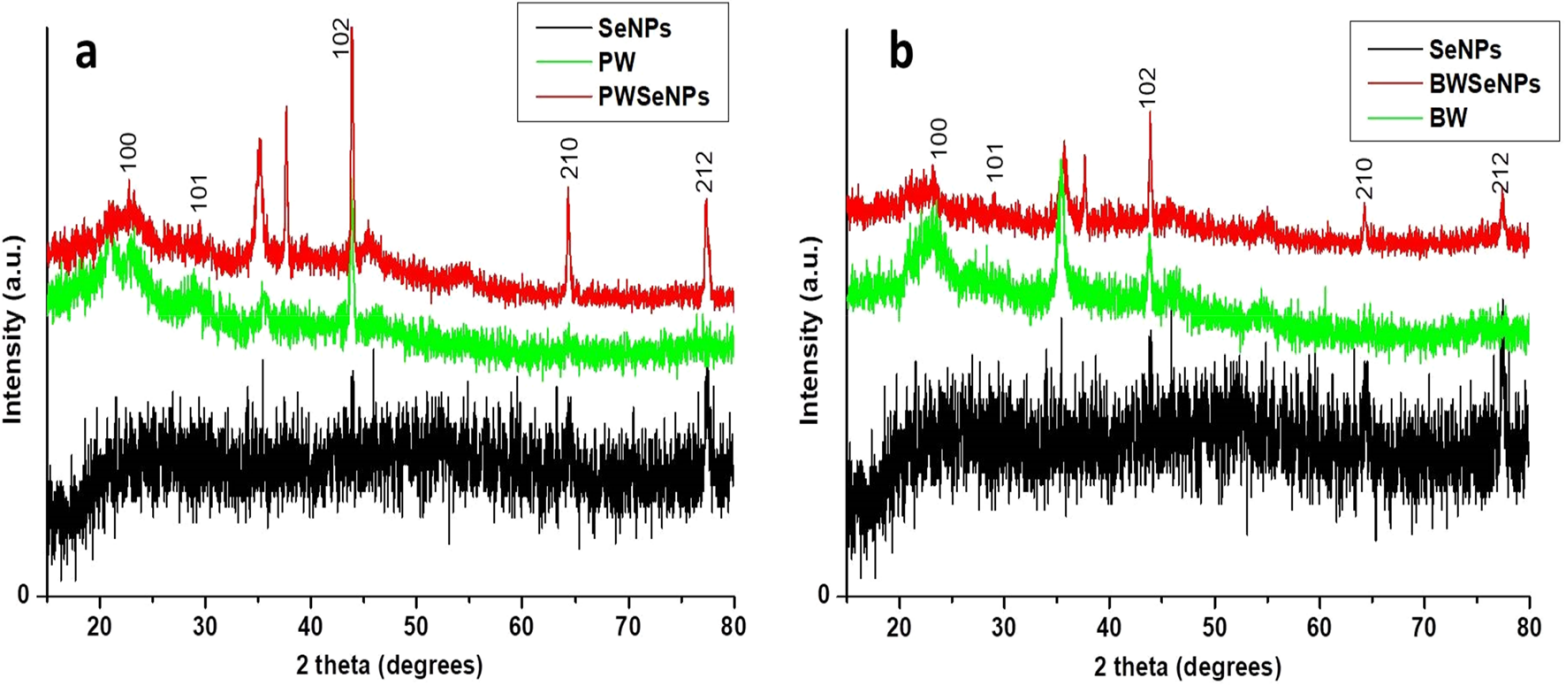
X-ray diffraction (XRD) spectra of (**a**) treated and untreated PW, and (**b**) treated and untreated BW comparing to Se-NPs spectrum.

XRD analysis was conducted to confirm the successful synthesis of the prepared Se-NPs and their deposition on the wood specimens impregnated with Se-NPs based on the XRD patterns and the crystallinity nature of Se-NPs. As displayed in Figure 4, the synthesized Se-NPs and those deposited on the wood specimens surfaces were highly crystalline. Additionally, the crystal planes of 100, 101, 102 and 210 observed at 24.28º, 29.24º, 43.64º and 64.28º, respectively, were attributed to Se-NPs, as described in the JCPDS 86-2246 international database^54^. All wood specimens impregnated with Se-NPs showed XRD patterns corresponding to Se-NPs, indicating the presence of the Se-NPs on their surfaces and the deposition of Se-NPs on the surfaces of the treated wood specimens.

#### Color measurements

**Figure 5:**
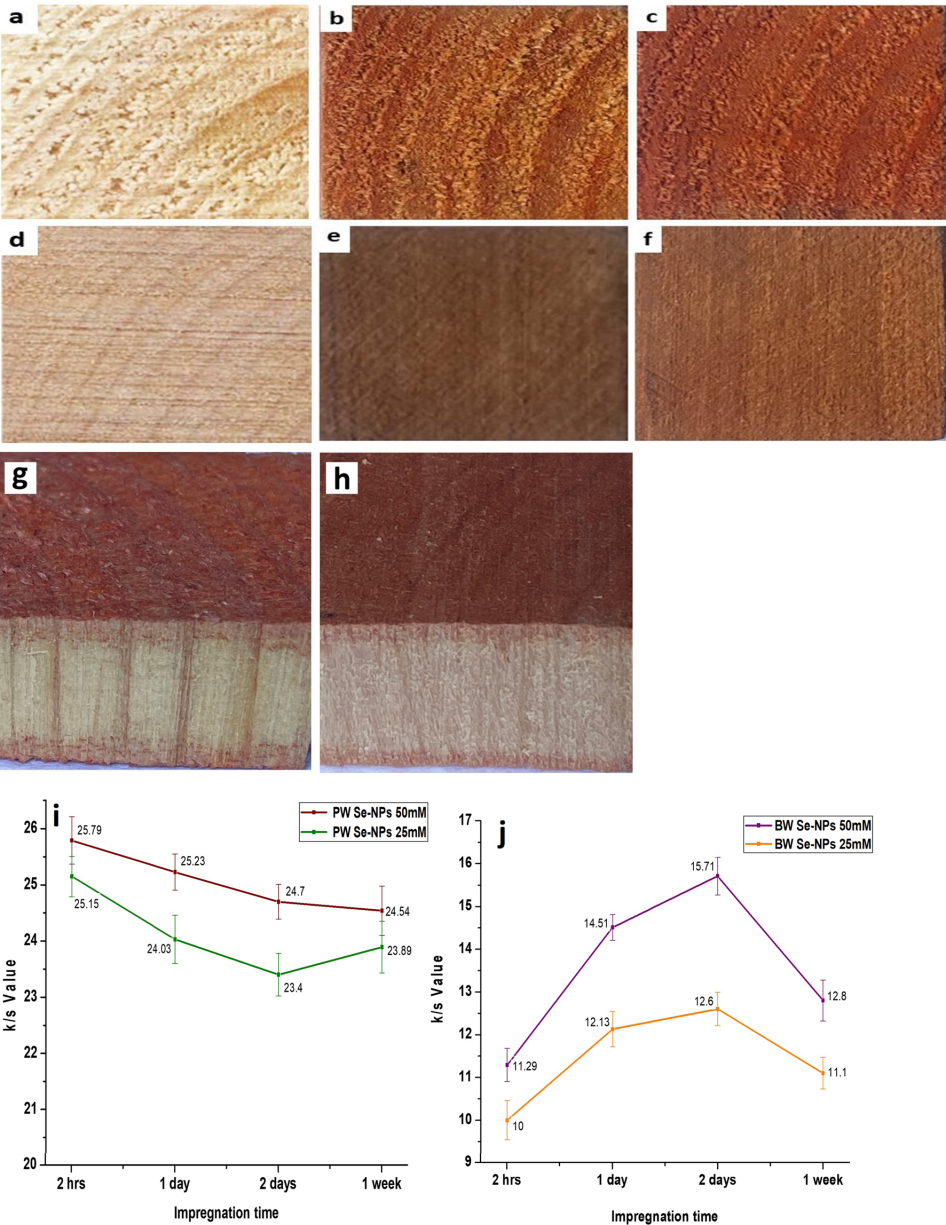
Images of PW surfaces: (**a**) blank, (**b**) treated with 25 mM Se-NPs, (**c**) treated with 50 mM Se-NPs, BW surfaces: (**d**) blank, (**e**) treated with 25 mM Se-NPs, (**f**) treated with 50 mM Se-NPs, penetration of 50 mM Se-NPs in (**g**) PW and (**h**) BW treated with 50 mM Se-NPs at the radial section, and the effect of Se-NPs concentration and impregnation time on K/S values of (**i**) the treated PW, and (**j**) the treated BW at 360 nm.

The colorimetric measurements of wood samples and those impregnated with Se-NPs were determined spectrophotometrically to study the effect of Se-NPs impregnation on the color properties of the PW and BW surfaces. The wood specimens impregnated with Se-NPs acquired a dark orange color as a result of treatment with Se-NPs. As shown in Figure 5, Se-NPs were deposited sufficiently on the surfaces of the treated wood specimens with good homogeneity. While, blank PW and BW samples were clear and free of NPs. The color intensity increased with increasing concentration of Se-NPs. The PW specimens absorbed higher amounts of NPs than BW specimens that may be due to the variation in their microstructure. BW has a permeability less than that of PW owing to the narrow pores in its surface^15^, as illustrated by the SEM micrographs in Figure 3.

The color strength (K/S) data of PW and BW wood were measured after impregnation with Se-NPs at 25 and 50 mM for various periods of 2 hrs, 1 day, 2 days, and 1 week, as shown in Figure 5. At low concentration of Se-NPs, the treated PW and BW samples exhibited low positive K/S values. With an increase in Se-NPs concentration, the K/S value increased and the treated samples gave a more reddish tone to the wood samples. The PW samples impregnated with Se-NPs exhibited higher K/S values than those of the treated BW, as shown in Figure 5. This comes to the fact that the BW fibers are generally shorter and smaller in diameter than the PW. In addition, the pits in the fibers of the BW are much smaller and less numerous, and hence less conspicuous than those in the PW^55,56^.

The highest K/S value was recorded for the PW specimens at an impregnation time of 2 hrs at a concentration of 50 mM Se-NPs. In addition, the color of the treated PW samples decreased as the impregnation time increased except for those impregnated with 25 mM Se-NPs for one week, which showed higher K/S values than those impregnated with the same concentration for 2 days. The decrease in the K/S value may be due to the aggregation of Se-NPs owing to the increase in the impregnation time and therefore the augmentation of the amount of Se-NPs that encourages NPs aggregation. While, the highest K/S values of the treated BW specimens were obtained after 2 days of impregnation. BW has less permeability than PW^15^; therefore, it absorbs fewer Se-NPs on the surface that means a low concentration of Se-NPs and slow aggregation over time on the surface, as shown in Figure 5.

#### Density and moisture content

Wood density is considered an important measurement and corresponds to several mechanical properties of wood^57^. The average density values of untreated and treated PW were lower than those of BW. This variation in density may be due to the difference in the microstructures of PW and BW. PW exhibits broader pores and higher permeability than BW^15^. The highest treated PW density values (0.426 and 0.434 g/cm3) were observed after impregnation periods of 2 days for those treated with 25 and 50 mM Se-NPs, respectively, and there were no significant variations in the density value between them and untreated PW, as listed in Table 1. However, the average density values of the other PW treated with Se-NPs were lower than the blank sample. Impregnation with 25 mM Se-NPs for 2 hrs and 1 day and with 50 mM for 1 day led to a significant decrease in PW density compared to the blank. This reduction in density may be due to the expansion of wood as a result of the impregnation process and water absorption^58^ that led to an obvious decrease in PW density. However, an increase in impregnation time may lead to the deposition of more Se-NPs onto the surface and an increase in density values^59^. In addition, the highest density values of treated BW were obtained for those impregnated with Se-NPs (25 mM and 50 mM) for only 2 hrs. Furthermore, there was a significant increase in the BW density as a result of the increase in the Se-NPs concentration at an impregnation time of 2 hrs. Moreover, no significant variations were observed among the mean density values of the other treated BW specimens and the blank sample. Overall, the density values of wood samples treated with 50 mM Se-NPs were usually higher than those treated with 25 mM Se-NPs. This variation may be due to the increase in the concentration of the deposited Se-NPs, which exhibit high density compared to the tested wood.

**Table 1:** Effect of impregnation with Se-NPs on the density and moisture contents of the treated wood and the depth of Se-NPs penetration into the treated PW and BW at both tangential and radial sides.

Wood type	Se-NPs Conc. (mM)	Impregnation time	Penetration depth (mm)		Moisture content (%)	Density (g/cm^3^)
			Tangential side	Radial side		
PW	Untreated	–	–	–	9.28 ± 0.062 g	0.428 ± 0.003 c
	25	2 h	3.34 ± 0.18 b	2.41 ± 0.11 bc	9.74 ± 0.106 b	0.380 ± 0.007 e
		1 day	2.46 ± 0.44 defg	2.12 ± 0.37 bcd	9.93 ± 0.032 a	0.391 ± 0.008 de
		2 days	3.90 ± 0.29 a	3.24 ± 0.67 a	9.86 ± 0.065 a	0.426 ± 0.038 c
		1 week	2.75 ± 0.61 cd	2.46 ± 0.26 bc	9.86 ± 0.022 a	0.414 ± 0.003 cde
	50	2 h	3.11 ± 0.12 bc	3.19 ± 0.60 a	9.30 ± 0.101 g	0.390 ± 0.025 de
		1 day	2.60 ± 0.39 cde	2.76 ± 0.34 ab	9.60 ± 0.007 c	0.413 ± 0.008 cde
		2 days	2.08 ± 0.27 efgh	2.36 ± 0.44 bc	9.76 ± 0.070 b	0.434 ± 0.036 c
		1 week	2.74 ± 0.17 cd	2.31 ± 0.43 bc	9.69 ± 0.041 b	0.415 ± 0.021 cd
BW	Untreated	–	–	–	8.45 ± 0.033 i	0.660 ± 0.011 b
	25	2 h	1.88 ± 0.43 ghi	1.40 ± 0.32 efg	9.39 ± 0.029 f.	0.684 ± 0.030 b
		1 day	2.50 ± 0.27 def	1.93 ± 0.37 cdefg	9.49 ± 0.033 de	0.669 ± 0.004 b
		2 days	1.98 ± 0.14 fghi	1.36 ± 0.39 fg	9.48 ± 0.008 de	0.670 ± 0.013 b
		1 week	2.19 ± 0.41 defgh	2.04 ± 0.40 bcdef	9.51 ± 0.016 d	0.673 ± 0.019 b
	50	2 h	2.40 ± 0.35 defg	2.10 ± 0.38 bcde	9.06 ± 0.004 h	0.725 ± 0.026 a
		1 day	2.67 ± 0.28 cde	2.41 ± 0.18 bc	9.33 ± 0.035 fg	0.676 ± 0.022 b
		2 days	1.74 ± 0.19 hi	1.59 ± 0.36 defg	9.37 ± 0.009 fg	0.674 ± 0.012 b
		1 week	1.41 ± 0.22 i	1.32 ± 0.08 g	9.40 ± 0.024 ef	0.661 ± 0.017 b

a–i Means within a column followed by the same letter(s) are not significantly different according to Duncan’s multiple range test (P = 0.05).

The moisture content of the treated and untreated wood was determined to study the effect of the Se-NPs concentration and impregnation time on the moisture content of the tested wood, as shown in Table 1. The moisture content of untreated BW was significantly lower than that of untreated PW, which has higher permeability and larger pores than BW^15,55^. In the case of PW, the moisture content increased significantly compared to the blank sample as a result of the impregnation process with Se-NPs, except for those impregnated with 50 mM Se-NPs for 2 hrs, which showed a non-significant increase in moisture content in comparison with the PW control. In general, the moisture content of PW and BW samples treated with 50 mM Se-NPs decreased significantly compared to those treated with 25 mM Se-NPs at the same impregnation time for the same wood type. This decrease may be due to the increase in the quantity of NPs deposited on the surface as the Se-NPs concentration increased, which may minimize the absorption of water. Therefore, impregnation with metal NPs such as Se-NPs is a relatively physical blocking that lowers the penetration of water into impregnated wood that enhances the physical and hydrophobic properties of wood treated with Se-NPs^60^.

#### Se-NPs penetration into wood

It was found that the depth of Se-NPs penetration into the treated wood varied based on the change in Se-NPs concentration, impregnation time, and the type of the tested wood. Regarding wood type, the average depth values of Se-NPs penetration into PW at both the tangential and radial sides were significantly higher than that of BW at the same impregnation time, as shown in Figure 5, except those impregnated with Se-NPs (25 and 50 mM) for 1 day or with 25 mM Se-NPs for 1 week, as shown in Table 1. BW exhibits less permeability than PW owing to its microstructure with narrow pores that may minimize Se-NPs penetration into the tested BW specimens^15,56^.

There were no significant variations in the penetration depth at the tangential side among the treated PW samples with the change in Se-NPs concentrations at the same impregnation time, except for those impregnated for 2 days. They showed a significant increase in Se-NPs penetration with the decrease in the Se-NPs concentration at the tangential side. In addition, similar results were obtained on the radial side, except for those treated with 25 mM Se-NPs for 2 hrs, which showed a significant increase in penetration depth with increasing Se-NPs concentration. This high penetration may be due to a high quantity of Se-NPs at the high concentration (50 mM) and the low impregnation time that prevents NPs aggregation compared to long impregnation periods. The obtained results indicated that relatively small Se-NPs in size were deposited well inside the wood compared to aggregated particles, which may be removed easily during the leaching process. No significant variations in penetration depth at both radial and tangential sides were observed among treated BW at the same impregnation period with the change in Se-NPs concentration except those impregnated for 1 week. They showed a significant decrease in the penetration depth as the increase in Se-NPs concentration, as listed in Table 1.

#### Antimicrobial activity

The antimicrobial activities of wood samples treated with Se-NPs were evaluated using the inhibition zone method according to the method described by Munir et al.^61^. Antimicrobial activities were tested against *Staphylococcus aureus*, *Escherichia coli*, *Candida albicans*, and *Bacillus cereus*. PW specimens impregnated with Se-NPs showed higher antimicrobial activities than those of treated BW samples due to the high permeability of PW (softwood) compared to that of BW (hardwood), which enables Se-NPs to penetrate the wood layers and increases the Se-NPs concentration inside it^55,56^. Moreover, Se-NPs concentration affected the antimicrobial activities of the treated wood specimens. In the case of PW, wood samples treated with high Se-NPs concentration (50 mM) showed higher antimicrobial activity than specimens soaked in Se-NPs colloidal solution at a concentration of 25 mM. On the other hand, BW samples treated with a low concentration of Se-NPs showed higher antimicrobial activity than specimens impregnated with a high concentration of Se-NPs. This may be due to the narrow pores in the case of BW, which obstruct Se-NPs permeability and result in Se-NPs accumulation on the wood surface with increasing soaking period. Therefore, Se-NPs aggregation leads to an increase in Se-NPs size, and therefore, decreases their antimicrobial activity. As shown in Figure 1, Se-NPs at a low concentration of 25 mM had small nanoparticles ranging from 30 to 80 nm, which enhanced their permeability through the BW surface and reduced the possibility of aggregation. Additionally, their hollow shape augmented their specific area and antimicrobial activities compared to those of Se-NPs prepared at 50 mM. The highest antimicrobial activity was obtained in the case of PW samples treated with 50 mM Se-NPs against *E. coli*. Furthermore, *S. aureus* showed higher sensitivity towards the decrease in Se-NPs diameters. The antimicrobial activity of wood specimens treated with Se-NPs against *S. aureus* increased with a decrease in Se-NPs size and an increase in soaking period in both PW and BW. On the other hand, *Candida albicans* was more resistant to the Se-NPs effect than the other tested microbes, as shown in Table 2.

**Table 2:** Effect of Se-NPs on the antimicrobial properties of the treated and untreated BW and PW samples.

Sample			Inhibition zone area (mm^2^)*			
Wood type	Se-NPs Conc. (mM)	Impregnation time	*Staphylococcus aureus*	*Bacillus cereus*	*Escherichia coli*	*Candida albicans*
PW	Untreated	–	0 ± 0.0 d	0 ± 0.0 d	0 ± 0.0 d	0 ± 0.0 d
	25	2 h	9 ± 0.5 h	10 ± 1.0 d	12 ± 0.3 e	0 ± 0.0 d
		1 day	12 ± 0.5 d	11 ± 1.2 bcd	19 ± 1.0 c	0 ± 0.0 d
		2 days	13 ± 0.3 cd	12 ± 0.5 bc	20 ± 0.6 b	0 ± 0.0 d
		1 week	15 ± 0.5 b	13 ± 0.8 a	22 ± 0.6 a	0 ± 0.0 d
	50	2 h	13 ± 0.5 c	10 ± 0.3 cd	10 ± 0.3 g	7 ± 0.9 c
		1 day	15 ± 0.5 a	11 ± 0.5 bc	11 ± 0.6 ef	11 ± 0.3 a
		2 days	17 ± 0.5 a	12 ± 0.3 ab	18 ± 0.3 d	10 ± 0.6 b
		1 week	13 ± 0.3 c	13 ± 0.5 ab	23 ± 0.6 a	11 ± 0.3 b
BW	Untreated	–	0 ± 0.0 d	0 ± 0.0 d	0 ± 0.0 d	0 ± 0.0 d
	25	2 h	4 ± 0.5 i	8 ± 0.5 ef	4 ± 0.0 j	0 ± 0.0 d
		1 day	9 ± 0.5 gh	8 ± 0.3 e	7 ± 0.5 i	0 ± 0.0 d
		2 days	17 ± 0.3 a	9 ± 0.5 e	11 ± 0.3 f.	0 ± 0.0 d
		1 week	12 ± 0.5 d	10 ± 0.3 d	11 ± 0.5 f.	0 ± 0.0 d
	50	2 h	9 ± 0.6 fgh	7 ± 0.5 f.	0 ± 0.0 k	0 ± 0.0 d
		1 day	10 ± 1.0 ef	8.5 ± 0.5 e	3 ± 0.5 j	0 ± 0.0 d
		2 days	11 ± 0.5 e	8 ± 0.5 ef	4 ± 0.5 j	0 ± 0.0 d
		1 week	10 ± 0.3 efg	9 ± 0.9 f.	8 ± 1.0 h	0 ± 0.0 d

a–k Means within a column followed by the same letter(s) are not significantly different according to Duncan’s multiple range test (P = 0.05).

## Conclusions

In this study, we succeeded in the impregnation of Se-NPs onto PW and BW surfaces to obtain antimicrobial wood with good color properties and high durability. The Se-NPs prepared at concentrations of 25 and 50 mM were characterized via instrumental identification, confirming the formation of Se particles at the nanoscale. The diameters of the Se-NPs decreased with increasing concentration, which also affected NPs morphology. At 25 mM, Se-NPs were spherical with a hollow shape, whereas Se-NPs synthesized at 50 mM were only spherical in shape. In addition, the prepared Se-NPs were impregnated at different concentrations for various periods onto the PW and BW surfaces to study the effect of Se-NPs concentration and impregnation time on the color and antimicrobial properties of the wood specimens. The wood samples impregnated with Se-NPs were examined using EDX, SEM, and XRD analyses, which illustrated successful Se-NPs deposition on the treated wood surfaces. The results of color properties revealed that the highest K/S values were obtained after impregnation with 50 mM Se-NPs for 2 hrs and 2 days of impregnation time in the case of PW and BW, respectively. The best antimicrobial activities were obtained after impregnation of Se-NPs into PW at a concentration of 50 mM for 1 week, and BW treatment with 25 mM Se-NPs for 2 days. PW and BW treatments using Se-NPs were helpful in improving the color and antimicrobial properties of the tested wood, thereby ensuring the applicability of Se-NPs as a protective and decorative layer for wood surfaces and enhancing wood durability. PW and BW, as soft- and hardwood species, are preferred and employed in the manufacturing of furniture, buildings, and interior and outdoor projects. In this respect, the increase in wood durability using Se-NPs treatment enhances the applicability of PW and BW in these fields.

### Supplementary Information

Supplementary Information 1.

Supplementary Information 2.

Supplementary Information 3.

Supplementary Information 4.

Supplementary Information 5.

Supplementary Information 6.

Supplementary Information 7.

Supplementary Information 8.

Supplementary Information 9.

Supplementary Information 10.

## Data Availability

All data generated or analyzed during this study are included in this published article [and its supplementary information files].
