# Supplementary material for: A novel approach for enhancing the color and antimicrobial properties of pine and beech wood using Se-NPs
Source: Sci Rep. 2023 Aug 10;13:12972. doi: 10.1038/s41598-023-39748-5 (PMC10415366; doi:10.1038/s41598-023-39748-5)
Supplement: Supplementary file 5 — Supplementary Information 5. [file 41598_2023_39748_MOESM5_ESM.pdf]

Project 1

9/21/2020 11:32:32 AM

Spectrum processing :
No peaks omitted

Processing option : All elements analyzed (Normalised)
Number of iterations = 5

Standard :
C CaCO3 1-Jun-1999 12:00 AM
O SiO2 1-Jun-1999 12:00 AM
Se Se 1-Jun-1999 12:00 AM

Elem...	Weight%	Atomic%
C K	53.10	60.73
O K	45.44	39.01
Se K	1.46	0.25
Totals	100.00	

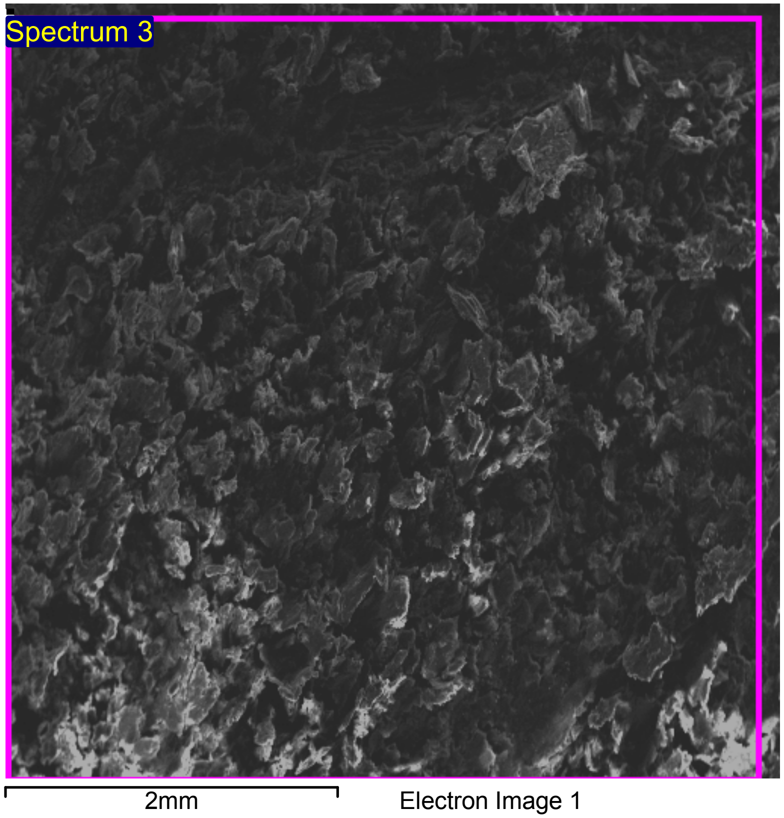

Electron Image 1

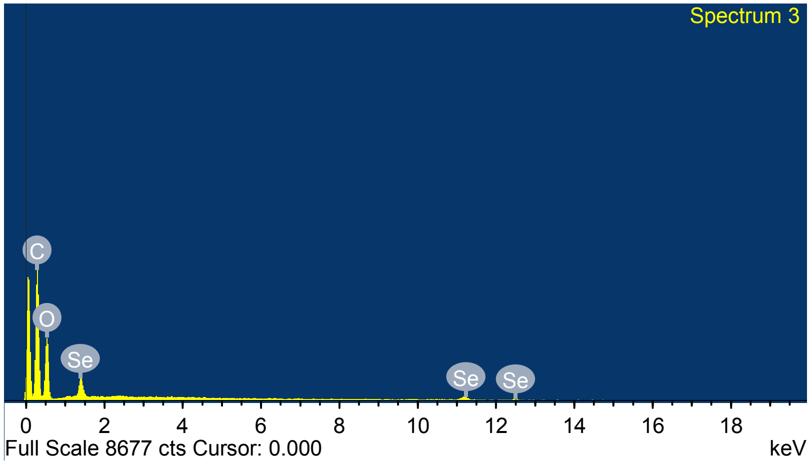

Comment:
