# Supplementary material for: A novel approach for enhancing the color and antimicrobial properties of pine and beech wood using Se-NPs
Source: Sci Rep. 2023 Aug 10;13:12972. doi: 10.1038/s41598-023-39748-5 (PMC10415366; doi:10.1038/s41598-023-39748-5)
Supplement: Supplementary file 6 — Supplementary Information 6. [file 41598_2023_39748_MOESM6_ESM.pdf]

Project 1

11/5/2019 12:55:33 PM

Spectrum processing :
Peaks possibly omitted : 2.146, 3.682, 9.692 keV

Processing option : All elements analyzed (Normalised)
Number of iterations = 5

Standard :
C CaCO3 1-Jun-1999 12:00 AM
O SiO2 1-Jun-1999 12:00 AM
Se Se 1-Jun-1999 12:00 AM

Elem...	Weight%	Atomic%
C K	51.93	59.31
O K	47.30	40.56
Se K	0.76	0.13
Totals	100.00	

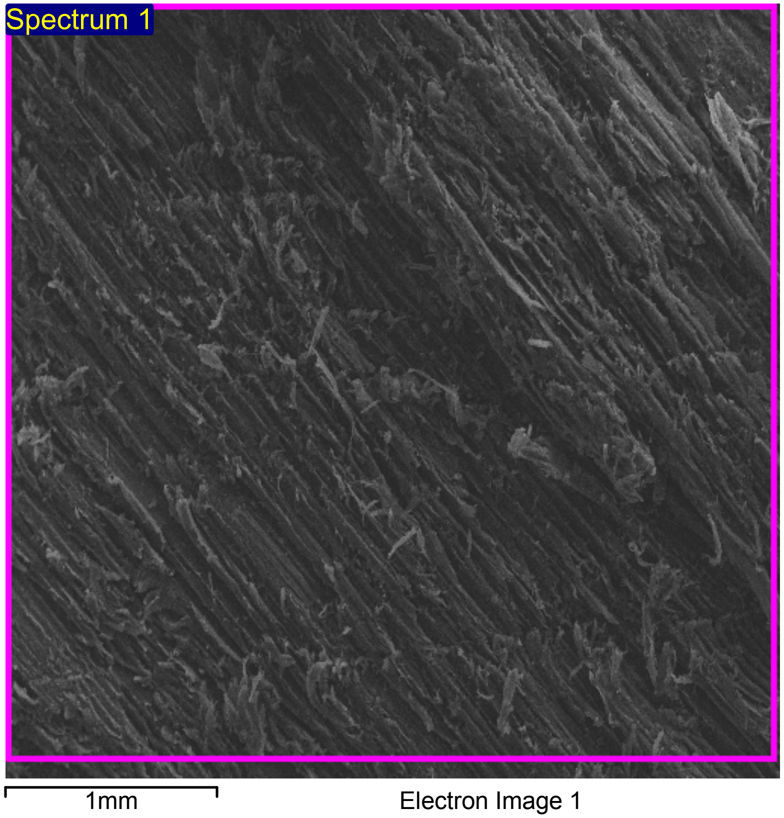

Electron Image 1

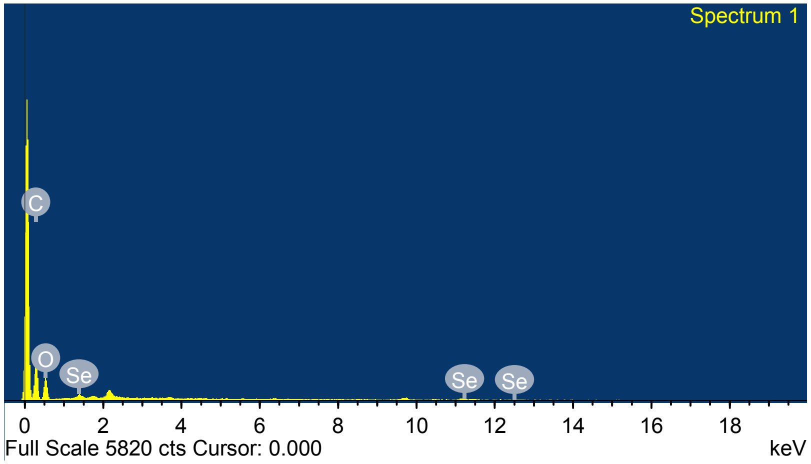

Comment:
